# Annexin A1 peptide and endothelial cell‐conditioned medium modulate cervical tumorigenesis

**DOI:** 10.1002/2211-5463.12603

**Published:** 2019-03-05

**Authors:** Laila Toniol Cardin, Janesly Prates, Bianca Rodrigues da Cunha, Eloiza Helena Tajara, Sonia Maria Oliani, Flávia Cristina Rodrigues‐Lisoni

**Affiliations:** ^1^ Institute of Bioscience, Humanities and Exact Science São Paulo State University (Unesp) São José do Rio Preto Brazil; ^2^ Department of Molecular Biology School of Medicine of São José do Rio Preto São José do Rio Preto Brazil; ^3^ School of Engineering São Paulo State University (Unesp) Ilha Solteira Brazil

**Keywords:** ANXA1, carcinogenesis, cervical cancer, inflammation, peptide treatment

## Abstract

Cervical cancer is one of the leading causes of cancer death in women worldwide, and its tumorigenesis can be influenced by the microenvironment. The anti‐inflammatory protein annexin A1 (ANXA1) has been reported to be associated with cancer progression and metastasis, suggesting that it plays a role in regulating tumour cell proliferation. Here, we examined the effect of the N‐terminal peptide Ac2‐26 of ANXA1 on the HaCaT cell line (normal) and HeLa cell line (cervical cancer) co‐cultured with endothelium cell‐conditioned medium (HMC). Treatment with Ac2‐26 decreased proliferation and increased motility of cervical cancer cells, but did not affect cellular morphology or viability. Combined HMC stimulus and Ac2‐26 treatment resulted in an increase in apoptotic HeLa cells, upregulated expression of *MMP2*, and downregulated expression of *COX2*,*EP3* and *EP4*. In conclusion, Ac2‐26 treatment may modulate cellular and molecular mechanisms underlying cervical carcinogenesis.

**Abbreviations**
Ac2‐26: peptide of ANXA1
ANXA1: annexin A1 protein
cAMP: cyclic adenosine monophosphate
COX2: cyclooxygenase 2
EP3: prostaglandin E receptor 3
EP4: prostaglandin E receptor 4
HMC: conditioned medium of HUVEC cells
HMCS: non‐treated conditioned medium of HUVEC cells
HMCT: treated conditioned medium of HUVEC cells
HPV: human papillomavirus
HUVEC: human umbilical vein endothelial cell
IL: interleukin
JNK: c‐Jun N‐terminal protein kinase
MAPK: mitogen‐activated protein kinase
MEM: minimum essential medium
MMP2: matrix metallopeptidase 2
MMP9: matrix metallopeptidase 9
PGE2: prostaglandin E2
TIMP1: TIMP metallopeptidase inhibitor 1

Cervical cancer is one of the main gynaecological cancers worldwide, and has been associated with the human papillomavirus (HPV) and/or infection with bacteria such as *Chlamydia trachomatis* and *Neisseria gonorrhoeae* 1, 2. Cervical neoplasm is associated with persistent HPV in more than 95% of cases, particularly in people with impaired immune system functions 2, 3. In overview, cervical cancer is the seventh most frequent type of tumour and fourth most common in women, disregarding non‐melanoma skin cancer 2, 4. Diagnosis may be ascertained using the Papanicolaou test, which assists in the identification of persistent inflammation caused by the HPV, and in cases of positive results, treatment may be initiated 5.

Besides infections, the cancer microenvironment also influences the tumour development process because it is very different from that of corresponding healthy tissue 6. The microenvironment has been characterised by its unsettled extracellular matrix composition, increased microvessel density, and abundance of inflammatory cells and fibroblasts with an activated phenotype 6, 7, 8. There is evidence that the cancer progression and development does not rely only on genetic characteristics, but also on the interaction between the cancer and stromal cells, including endothelial cells, immune cells, fibroblasts, adipocytes and inflammatory cells 6. The different tumour phases that are associated with inflammation are considered an imminent threat, directly connected to the development process 9. In the inflammatory scenario, Annexin A1 (ANXA1) has anti‐inflammatory effects and therefore plays a key role in the modulation of the inflammatory response.

Annexin A1, which is also expressed by the tumour cells, acts as a modulator of the inflammatory process and has been linked to tumourigenesis 10. Studies involving ANXA1 and cancer are controversial, but nevertheless indicate that this protein may be a target for new therapeutic interventions and used as a potential biomarker 11. Its functions are specific to each type of cancer and there is evidence to indicate that its regulation and subcellular localisation are linked to the development, invasion, metastasis, progression and treatment resistance of tumours 12, 13, 14, 15.

Structurally, the protein has a C‐terminal core, representing 80% of its composition; this portion is common to all members of the annexin superfamily, and has four repeated homologous sequences and the ‘type 2’ domain for calcium linkage 11. The variable N‐terminal core is unique in length and sequence to each member of the family, and includes potential phosphorylation, glycosylation and peptidase action sites 16, 17.

The biological activity of ANXA1 can be reproduced by the first 26 amino acids of the N‐terminal core 18 or by some smaller peptides 19. Since this was discovered, it has become common practice to use these molecules in experimental models of acute 20, 21, 22, 23, chronic 24 and systemic inflammation 25, and also in *in vitro* studies using different cancer cell lines 26, 27, 28, 29. Recently, the use of the peptide was evaluated in skin allograft 30 and in inflammatory ocular disease 21, 31.

There is evidence of a relationship between ANXA1 expression and cervical tumourigenesis. To ascertain the upregulation of the phosphorylated protein according to disease progression, samples from dysplasia and cervical cancer stages I, II, and III have been used 32. Other work showed that ANXA1 was downregulated in all stages of the disease 33, and another study, analysing healthy, stage I, II and III, and invasive cancer samples, demonstrated that the protein expression levels corresponded to the disease progression 34.

ANXA1's contributions to tumourigenesis are still not well known, and considering its role in inflammation, it is an important area of research. The available data also point to controversies in the expression of this protein in cervical carcinogenesis, indicating a possible research field.

Considering the important role of ANXA1 in the inflammatory response and in tumours, we analysed the activity of the synthetic peptide of the ANXA1 protein in a cervical carcinoma cell line, along with the conditioned medium of endothelial cells, to help elucidate the processes that occur in the tumour microenvironment and expand understanding of ANXA1 as a therapeutic alternative. The rationale for this co‐treatment is that paracrine factors in the conditioned medium of human umbilical vein endothelial cells (HUVECs) simulate the cancer microenvironment, which influences the tumour development process, and is very different from that of corresponding healthy tissue.

## Results

### Ac2‐26 peptide response

**Figure 1:**
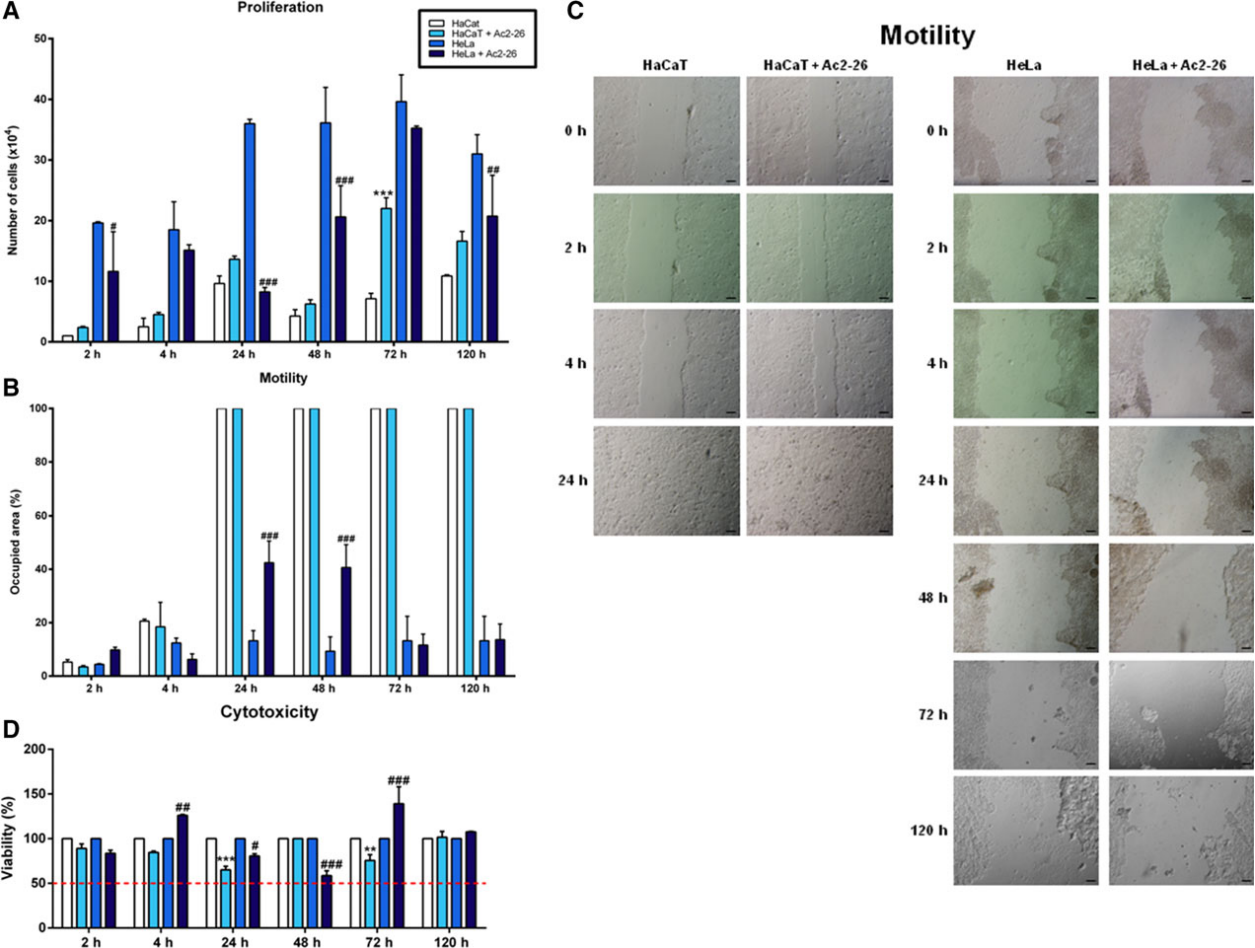
Response to Ac2‐26 peptide treatment of HaCaT and HeLa cell lines in proliferation, motility and cytotoxicity assays. The cells were cultured in complete minimum essential medium (MEM) and treated with Ac2‐26 (10 μg·mL^−1^). (A) Proliferation; (B) motility; (C) images illustrating motility; (D) cytotoxicity. *P* < 0.05 was considered significant; one symbol, *P* < 0.05; two symbols, *P* < 0.01; three symbols, *P* < 0.001: * *vs* HaCaT, # *vs* HeLa; ANOVA followed by Bonferroni's test. Assays were performed with three independent experiments. Error bars indicate SD. Scale bars: 500 μm.

**Figure 2:**
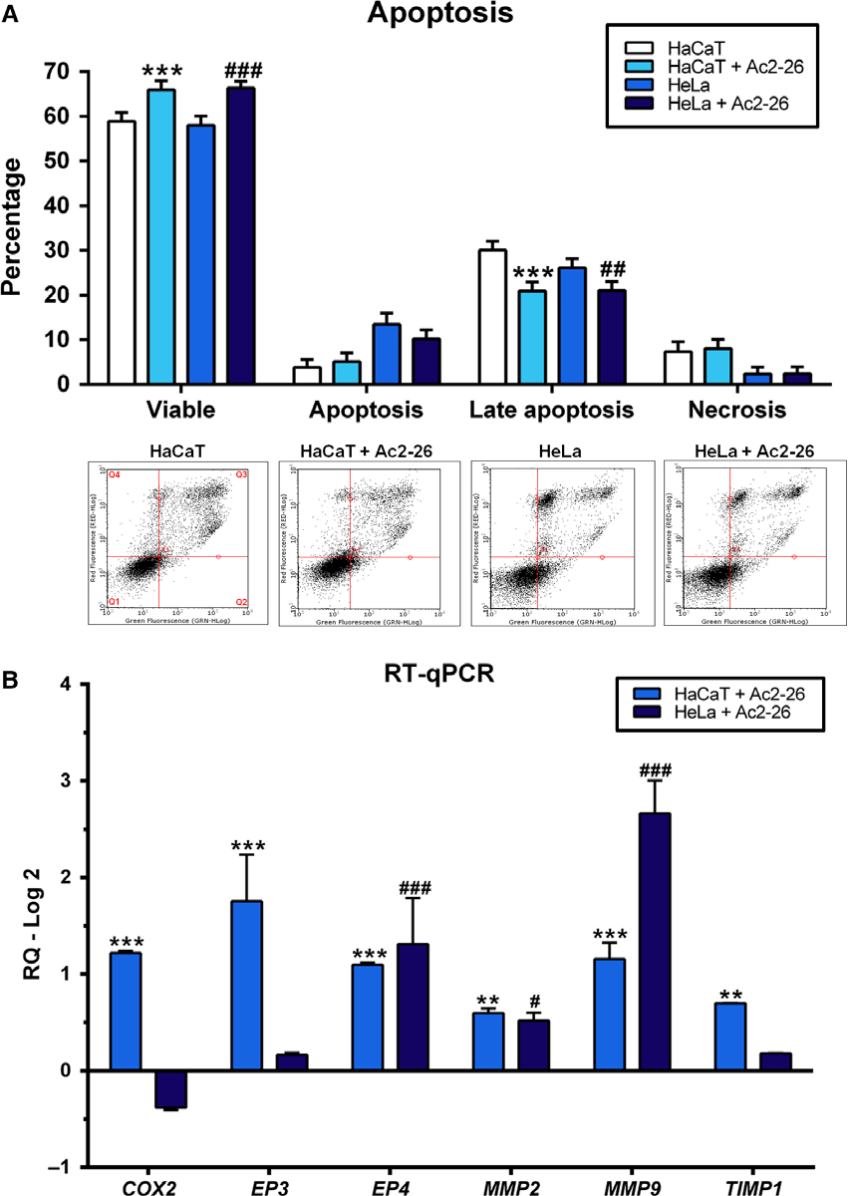
Response to Ac2‐26 peptide treatment by HaCaT and HeLa cell lines in an apoptosis assay and gene expression. The cells were cultured in complete MEM and treated with Ac2‐26 (10 μg·mL^−1^). (A) Densitometry and DotBlot apoptosis; *y* = cell percentage in 10 000 events. (B) Gene expression. *P* < 0.05 was considered significant; one symbol, *P* < 0.05; two symbols, *P* < 0.01; three symbols, *P* < 0.001: * *vs* HaCaT; # *vs* HeLa; ANOVA followed by Bonferroni's test. Assays were performed with three independent experiments. Error bars indicate SD.

Proliferation, motility and cytotoxicity of the human immortalised keratinocyte (HaCaT) cell line and the HeLa cell line (human cervical adenocarcinoma cells infected with HPV18) in response to Ac2‐26 peptide treatment were studied. The HaCaT cell line showed an increase in proliferation after 72 h (Fig. 1A), and motility after 24 h, closing the experimental wound, and for this reason the cells detached from the well plate, after 24 h (Fig. 1B and C). In the HeLa cell line, proliferation was decreased after 2, 24, 48 and 120 h (Fig. 1A), while motility was increased after 24 and 48 h (Fig. 1B). Cytotoxicity was not observed in either cell line at any of the experimental times (Fig. 1D). Late apoptosis was decreased in both cell lines after the treatment (Fig. 2A). Gene expression showed an upregulation of all six genes analysed in the HaCaT cell line, and of prostaglandin E receptor 4 (*EP4*), matrix metallopeptidase 2 (*MMP2*) and matrix metallopeptidase 9 (*MMP9*) in the HeLa cell line (Fig. 2B).

### Conditioned medium of endothelial cells (HMC) and Ac2‐26 peptide response

**Figure 3:**
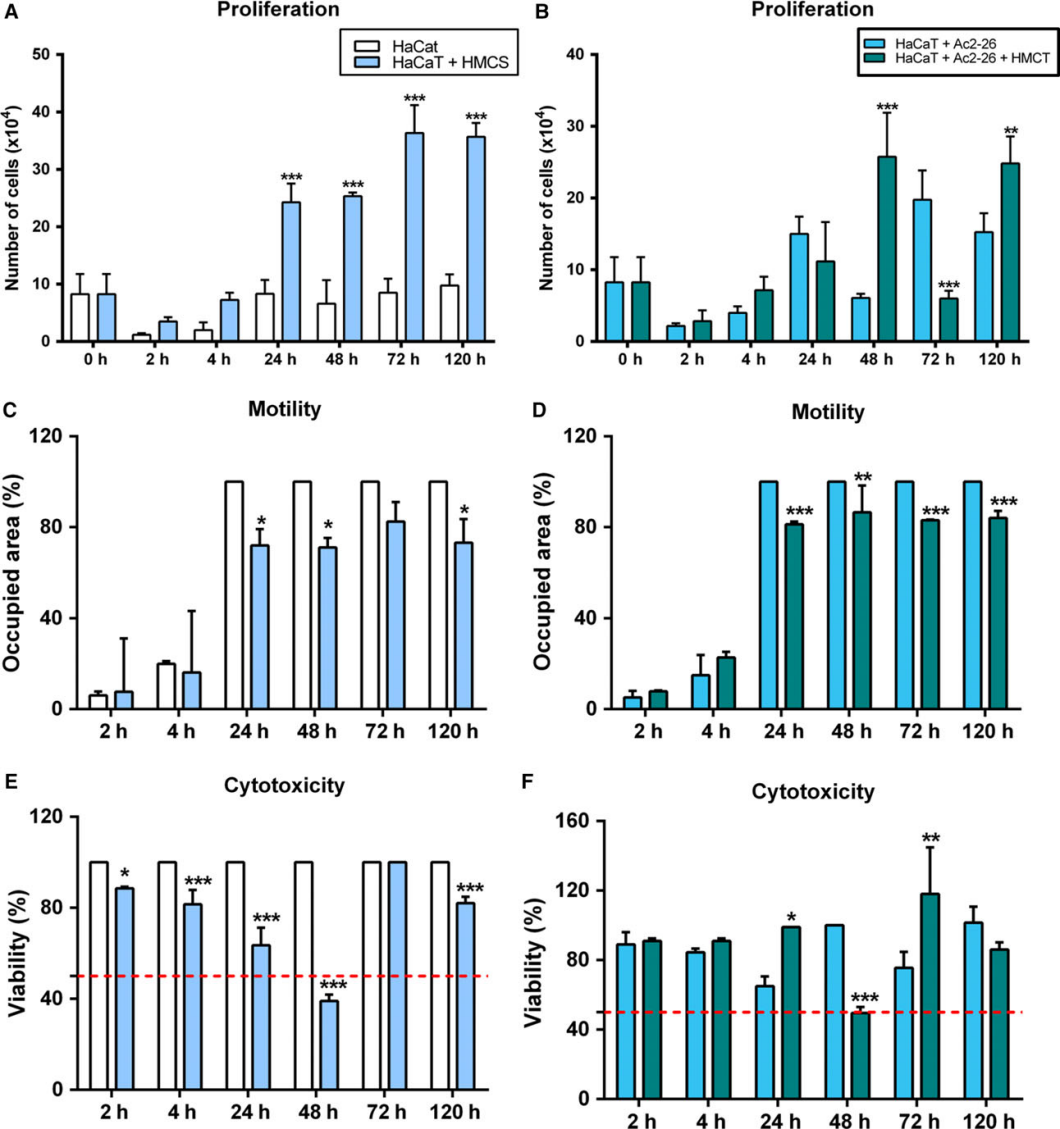
Response of the HaCaT cell line to conditioned medium induction and Ac2‐26 peptide treatment. The cells were cultured in complete MEM and stimulated with conditioned HUVEC cell medium (HMC) (at a ratio of 1 : 1) that was untreated (HMCS; A,C,E) or treated (HMCT; B,D,F) with Ac2‐26 (at 10 μg·mL^−1^). (A,B) HaCaT proliferation; (C,D) HaCaT motility; (E,F) HaCaT cytotoxicity. *P* < 0.05 was considered significant; *P < 0.05; **P < 0.01; ***P < 0.001: (A,C,E) *vs* HaCaT; (B,D,F) *vs* HaCaT + Ac2‐26; ANOVA followed by Bonferroni's test. Assays were performed with three independent experiments. Error bars indicate SD.

In the HaCaT cell line, secreted factors from endothelial cells (HUVECs) without Ac2‐26 peptide treatment (HMCS) increased proliferation after 24 h (Fig. 3A). With the combination of secreted factors of endothelial cells and Ac2‐26 treatment (HMCT), it was possible to observe an increase of the proliferation at 48 and 120 h, but a decrease at 72 h (Fig. 3B). Motility decreased after 24 h in the HaCaT cells (Fig. 3C,D) after induction with the conditioned medium without (HMCS) and with (HMCT) Ac2‐26 peptide treatment. Moreover, both conditions showed cytotoxicity to these cells only at 48 h (Fig. 3E,F).

**Figure 4:**
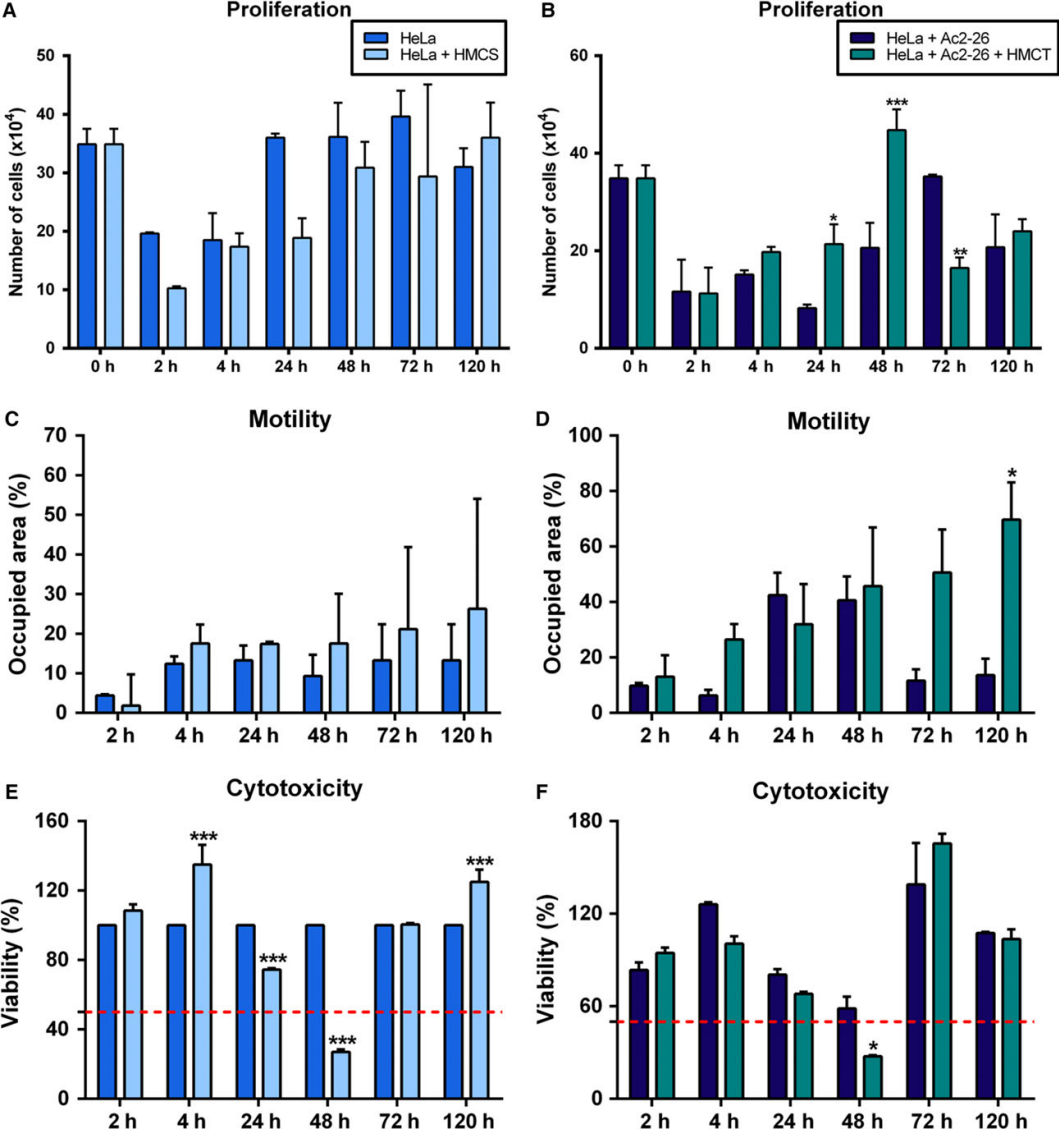
Response of the HeLa cell line to conditioned medium induction and Ac2‐26 peptide treatment. The cells were cultured in complete MEM and stimulated with conditioned HUVEC cell medium (HMC) (at a ratio of 1 : 1), untreated (HMCS; A,C,E) or treated (HMCT; B,D,F) with Ac2‐26 (at 10 μg·mL^−1^). (A,B) HeLa proliferation; (C,D) HeLa Motility; (E,F) HeLa cytotoxicity. *P* < 0.05 was considered significant; *P < 0.05; **P < 0.01; ***P < 0.001: (A,C,E) *vs* HeLa (B,D,F) *vs* HeLa + Ac2‐26; ANOVA followed by Bonferroni's test. Assays were performed with three independent experiments. Error bars indicate SD.

In the HeLa cell line, the secreted factors of endothelial cells without treatment (HMCS) led to a decrease in proliferation after 24 h (Fig. 4A), while with induction with HMC and the peptide treatment there was a decrease in proliferation at 72 h, but an increase at 24 and 48 h (Fig. 4B). In HeLa cells, motility had increased at 4 h after the induction with the conditioned medium without and with the treatment (HMCS and HMCT), but at 24 h there was a decrease after HMCT induction that only became statistically significant in 120 h (Fig. 4C,D). As with the HaCaT cell line, HeLa cells showed cytotoxicity only at 48 h (Fig. 4E,F).

**Figure 5:**
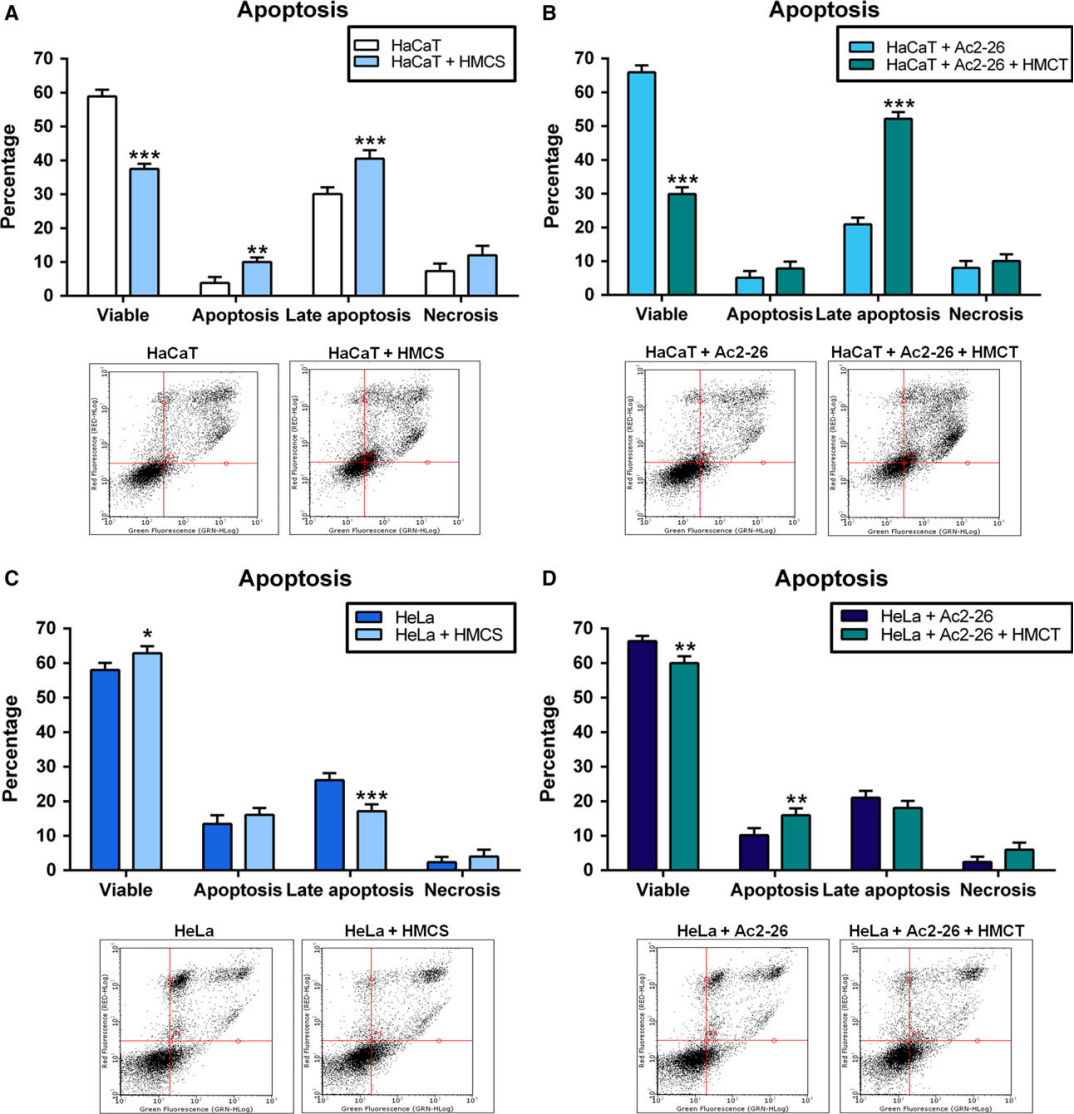
Response of HaCaT and HeLa cell lines to conditioned medium induction and Ac2‐26 peptide treatment. The cells were cultured in complete MEM and stimulated with conditioned HUVEC cell medium (HMC) (at a ratio of 1 : 1), untreated (HMCS; A–C) or treated (HMCT; B–D) with Ac2‐26 (at 10 μg·mL^−1^). (A,B) HaCat densitometry and DotBlot apoptosis; (C,D) HeLa densitometry and DotBlot apoptosis; *y* = cell percentage in 10 000 events. *P* < 0.05 was considered significant; *P < 0.05; **P < 0.01; ***P < 0.001: (A–C) *vs* Control of each group; (B–D) *vs* Control + Ac2‐26 of each group; ANOVA followed by Bonferroni's test. Assays were performed with three independent experiments. Error bars indicate SD.

Late apoptotic cells were increased in the HaCaT cell line (Fig. 5A,B) after induction with the conditioned medium without and with the treatment (HMCS and HMCT), but decreased in the HeLa cells (Fig. 5C) after induction with the conditioned medium without the treatment (HMCS). In the HeLa cells after the induction with the conditioned medium with the treatment (HMCT), it was possible to observe an increase in apoptotic cells, but still showing more viable cells (Fig. 5C,D).

**Figure 6:**
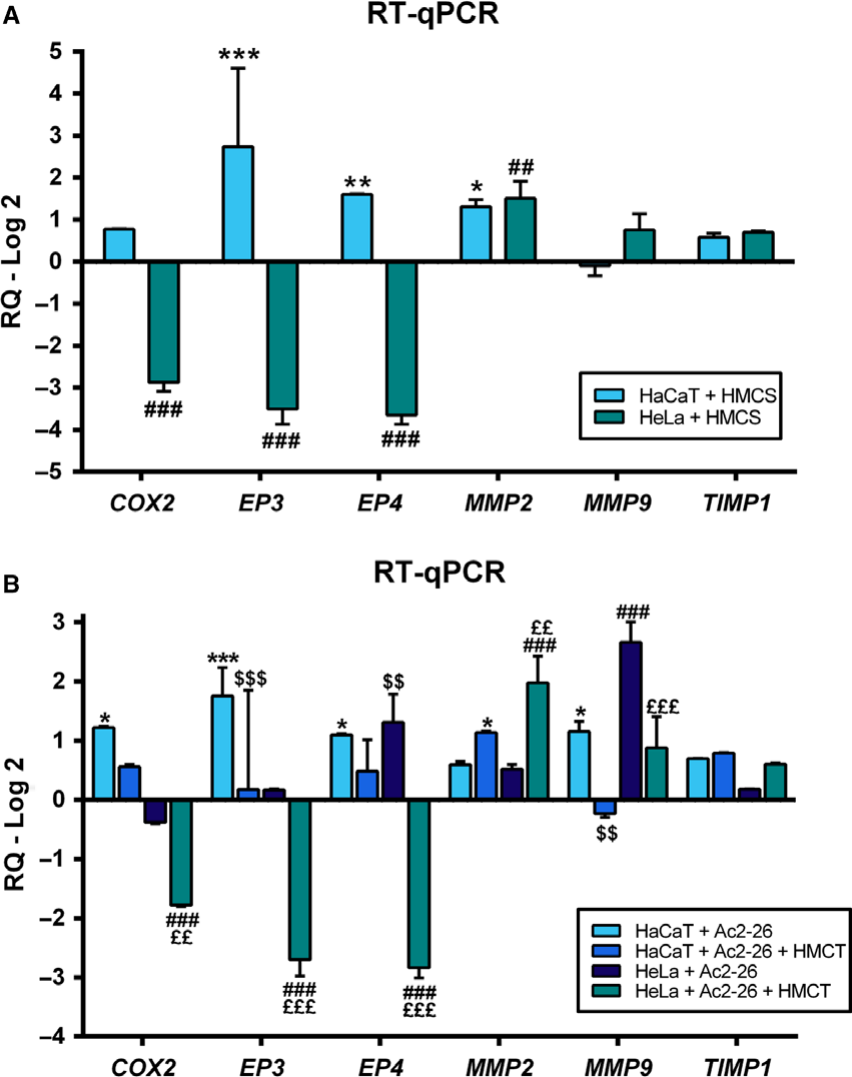
Response of HaCaT and HeLa cell lines to conditioned medium induction and Ac2‐26 peptide treatment. The cells were cultured in complete MEM and stimulated with conditioned HUVEC cell medium (HMC) (at a ratio of 1 : 1) untreated (HMCS; A) or treated (HMCT; B) with Ac2‐26 (at 10 μg·mL^−1^). (A) Gene expression after HMCS induction. (B) Gene expression after Ac2‐26 treatment and HMCT induction. *P* < 0.05 was considered significant; one symbol, *P* < 0.05; two symbols, *P* < 0.01; three symbols, *P* < 0.001: (A) * *vs* HaCaT; # *vs* HeLa; (B) * *vs* HaCaT; $ *vs* HaCaT + Ac2‐26; # *vs* HeLa; £ *vs* HeLa + Ac2‐26; ANOVA followed by Bonferroni's test. Assays were performed with three independent experiments. Error bars indicate SD.

The secreted factors of endothelial cells without the peptide treatment (HMCS) upregulated prostaglandin E receptor 3 (*EP3*), *EP4* and *MMP2* gene expression in the HaCaT cell line, while downregulating cyclooxygenase 2 (*COX2*), *EP3* and *EP4* and upregulating *MMP2* gene expression in the HeLa cell line (Fig. 6A).

The peptide treatment upregulated *COX2*,* EP3*,* EP4* and *MMP9* gene expression in the HaCat cell line and also upregulated *EP4* and *MMP9* gene expression in the HeLa cell line. The combination of the secreted factors from endothelial cells with Ac2‐26 treatment upregulated *MMP2* gene expression in HaCat cells, and downregulated *COX2*,* EP3* and *EP4* gene expression, but upregulated *MMP2* gene expression in HeLa cells (Fig. 6B).

## Discussion

Cervical cancer is related to HPV infection, and microbial infection is associated with 20–25% of the cancer cases worldwide 35. It has been observed that the start of the carcinogenesis is more associated with persistent inflammation, as a result of infections or autoimmunity 36. Besides the infections, the tumour microenvironment also plays a role in the tumourigenesis, due to its complex structure, in which different factors may influence and modify the cancer 37, 38, 39, and our experiments demonstrate this heterogeneity.

Our results demonstrate that cervical cancer cells exhibit a decrease in growth when treated with the Ac2‐26 peptide, and are stimulated by the conditioned medium of endothelial cells. This is important for our understanding of the role of this protein in cancer. These conditions also result in an increase in motility after 120 h, and the maintenance of viability, the stimulation becoming cytotoxic only at 48 h. Regarding apoptosis and gene expression, an increase in apoptotic cells, the downregulation of *COX2*,* EP3* and *EP4*, and the upregulation of *MMP2* were observed.

The pro‐ and anti‐inflammatory effects of ANXA1, a protein known to be an endogenous glucocorticoid, depend on the type of ligand, and have been described previously 12. ANXA1 protein was demonstrated to initiate the p38 mitogen‐activated protein kinase (MAPK) pathway and the Ac2‐26 peptide promotes the initiation of the c‐Jun N‐terminal protein kinase (JNK) pathway 40. The MAPK family pathway is one of the most important cascades responsible for reading an extracellular stimulus and unleashing a response in the cells, and one of these pathways is the JNK pathway, a signalling cascade known to be activated by stress 41. The JNK cascade may act by phosphorylating different molecules, among them p53 42. Through this action, we could suggest that the proliferation decrease after the Ac2‐26 treatment and HMC stimulation may be through activation of the MAPK family and targeting of p53, resulting in cell cycle arrest.

The proliferation decrease found in this study is corroborated by refs 26, 27, which also observed this phenomenon in cancer cell lines after treatment with ANXA1. One of the authors indicated that ANXA1 may act as a tumour suppressor, possibly acting on the extracellular signal‐regulated kinases 1 and 2 pathway, also a signalling cascade from the MAPK family 27. This response may result in overexpression of this pathway, which modifies the expression of the molecules involved in the cell cycle, resulting in arrest in the G1/S phase 43. This adds to the suggestion that the ANXA1 protein may have an anti‐proliferative role in the cervical cancer cells through the MAPK family pathway, as observed after the peptide treatment and stimulation with HMC.

The cytotoxicity assay demonstrated that the cells were as viable as the healthy control cells. Therefore, although the proliferation decreased after stimulation with the HMC and peptide treatment, the cells remained viable, making it possible to proceed with the cellular processes. There is an association between nuclear factor‐κB and ANXA1, suggesting that Annexin A1 protein activates this pathway promoting metastasis 44, which could be a result of the maintenance of motility.

The apoptosis analysis showed that the majority of the cancer cells were viable, but some were in apoptosis and late apoptosis. The cervical cancer apoptosis may be altered because of HPV18 infection. This virus has E6 and E7 domains, which express oncoproteins, and they are involved with *p53* and retinoblastoma degradation 45, 46, 47. The E6 protein, specifically, can form a complex that causes p53 polyubiquitination and its degradation by the proteasome 45, 48. The *p53* gene is also associated with the control of apoptosis by regulating the expression of two genes, B‐cell lymphoma 2 (*BCL2*; anti‐apoptotic) and BCL2‐associated X apoptosis regulator (*BAX*; pro‐apoptotic) 49. ANXA1 has a role relating to apoptosis regulation according to 11, and some research groups suggest the involvement of this protein as a pro‐apoptotic factor, whereas other groups indicate its role as an anti‐apoptotic factor. The activation of the MAPK pathways could also have an important role in the apoptotic process, and a range of anti‐cancer substances activate the MAPK cascade with the aim to promote apoptosis of the cancer cells 41. Although there were a greater number of viable cells, we could observe an increase in apoptotic cells after the peptide treatment and stimulation with HMC.

There are studies with ANXA1 showing its pro‐ and anti‐inflammatory responses and also associating its expression with metastasis 50. During the invasion process, it is suggested that a cell population, called ‘leader’ cells, invade the adjacent matrix, opening space for following cells 51. It is necessary that the ‘leader’ cells secrete factors in order to initiate the process, which will provide a path for the other cells; one of these factors is matrix metalloprotease 52. Boudhraa *et al*. 53 have observed that ANXA1 shows expression patterns related to specific tumour types, and they have associated the protein cleavage with melanoma cell aggressiveness. The same group showed that in melanoma cells, the administration of Ac2‐26 peptide activates formyl peptide receptors and *MMP2* gene expression 54. In our current work, the level of *MMP9* gene expression equalled the control, after HMC stimulation and peptide treatment, and the upregulation of *MMP2* was also observed in the healthy HaCaT cells; therefore this pattern could be due to the secreted paracrine factors of the endothelium, possible as an attempt to restore a healthy environment.

Pro‐carcinogenic functions have been attributed to the *COX2* gene, supporting the strategy of gene suppression; some of the processes related to *COX2* are apoptosis inhibition, proliferation increase, and induction of angiogenesis 55, 56, 57. The available literature shows that this gene may be triggered by different factors, for instance, bacterial lipopolysaccharide, interleukin (IL)‐1β, IL‐2, tumour necrosis factor α, epidermal growth factor and transforming growth factor β; and it may be blocked by anti‐inflammatory factors, such as corticosteroids, IL‐13, IL‐10 and IL‐4 55, 58, 59. In this work, it was possible to observe that the peptide treatment and HMC stimulation downregulated *COX2* gene expression in the cervical cancer cells. Prostaglandin E2 (PGE2) is one of the most abundant COX2 products due to the fact that PGE2 exists in all cell types 55. Besides stimulation of pain and inflammation, PGE2 participates in proliferation, apoptosis, and metastasis mechanisms 60, 61. PGE2 initiates its actions through specific G‐protein‐coupled membrane receptors, namely EP1, EP2, EP3 and EP4, with each of them initiating a specific cellular pathway 62. The EP2 and EP4 receptors stimulate adenylate cyclase to increase cyclic adenosine monophosphate (cAMP) production, a glycogen synthase kinase‐3 activator involved in the cell cycle 55. cAMP promotes β‐catenin phosphorylation and degradation by the proteasome 26S, stopping the cell cycle 55. The EP3 receptor has the opposite action to that described above; its interaction with PGE2 leads to the downregulation of adenylate cyclase and cAMP decrease 60.

The culture of HeLa cells with HMC stimulation showed that the treatment did not alter cell motility, but it did result in a decrease in cell proliferation, and in *COX2*,* EP3* and *EP4* gene expression. The remaining production of PGE2 could be acting through the EP4 receptor, culminating in a decrease of cellular growth after treatment, which could be interfering with apoptosis, since a lower number of cells was observed in this cellular process.

Our research studied for the first time the interaction of ANXA1 with the conditioned medium of endothelial cells, an attempt to simulate the tumour microenvironment. It has been possible to observe the likely existence of a complex interaction, and the diverse array of secreted factors may influence cellular and molecular mechanisms in different ways. The diverse types of tumours exhibit different biological roles and signalling pathways when ANXA1 is involved, and this contradiction is explained by the differential expression patterns and biological behaviours of this protein 11. In addition, calcium binding to ANXA1's C‐terminal core, which binds to the phospholipids, may determine the expression specificity and promote its functional diversity 16.

We suggest that along with the tumour cell secreted paracrine factors, ANXA1 diminishes proliferation and does not alter the cellular cytotoxicity. It was possible to observe an increase in apoptotic cells and a downregulation of *COX2*,* EP3* and *EP4*. Altogether, these data provide further insight into the protective effect of ANXA1 and its mimetic peptides in cervical tumourigenesis, and more research needs to be carried out to confirm its use.

## Materials and methods

### Cell culture

Three cell lines were used, HUVECs, HaCaT and HeLa cells. The HUVEC cell line was cultured in MEM/Earle medium (Cultilab, Brazil), pH 7.5, supplemented with 10% fetal bovine serum (Cultilab, Campinas, SP, Brazil), 1% antibiotic/antimycotic (Invitrogen, Carlsbad, CA, USA), and 1% l‐glutamine (200 μm) (Sigma‐Aldrich, St Louis, MO, USA). The HaCaT cell line was cultured in MEM/Earle medium (Cultilab), pH 7.5, supplemented with 10% fetal bovine serum (Cultilab), 1% antibiotic/antimycotic (Invitrogen), 1% l‐glutamine (200 μm) (Sigma‐Aldrich), 1% non‐essential amino acids (10 mm; Sigma‐Aldrich), and 1% sodium pyruvate (100 mm; Sigma‐Aldrich). The HeLa cell line were cultured in MEM/Earle medium (Cultilab), pH 7.5, supplemented with 10% fetal bovine serum (Cultilab), 1% antibiotic/antimycotic (Invitrogen) 1% l‐glutamine (200 μm; Sigma‐Aldrich), and 1% non‐essential amino acids (10 mm; Sigma‐Aldrich). A total of 10^6^ cells from each cell line were seeded in 75 cm^2^ culture flasks and kept at 37 °C in an atmosphere of 5% CO_2_.

We did all the experiments with another cervix cell line (SiHa – ATCC, Manassas, VA, USA) treated with the peptide, but without the co‐treatment with conditioned medium of endothelial cells (HUVECs), and the results were the same with the HeLa; for this reason we did not continued with the co‐treatment.

### Cellular co‐culture

The co‐culture was performed with the conditioned medium of the HUVEC (HMC) cell line, which was used in the co‐cultures with the other two cell lines. The HMC was obtained after HUVEC culture when the cells had acquired 80% confluence. The medium (HMC) was collected after 24 h of culture, from the HUVEC cell line medium without treatment (HMCS) and with treatment (HMCT). The treatment was performed with the N‐terminal Ac2‐26 peptide of ANXA1 (Ac‐MVSEFLKQAWFIENEEQEYVQTVK) 63, at a concentration of 10 μg·mL^−1^ 27. The co‐culture was performed at a 1 : 1 dilution, according to Rodrigues‐Lisoni *et al*. 64.

### Pharmacological treatment

The cell lines were cultured in complete medium, as described above, and subsequently, submitted for co‐culture with HMC and treated with the N‐terminal Ac2‐26 peptide of ANXA1 (Ac‐MVSEFLKQAWFIENEEQEYVQTVK) 63, at a concentration of 10 μg·mL^−1^ for six different time points (2, 4, 24, 48, 72 and 120 h) to perform the proliferation, wound healing, and cytotoxicity assays. After the analysis of these experiments one time point was chosen to continue the other assays. The experimental groups are described below:

**Table:** 

HaCaT	HeLa
HaCaT + Ac2‐26	HeLa + Ac2‐26
HaCaT + HMCS	HeLa + HMCS
HaCaT + Ac2‐26 + HMCT	HeLa + Ac2‐26 + HMCT

This study was performed to analyse the response of Ac2‐26 peptide treatment in cancer cell line, and in that way we developed the experiments only with the cells and the peptide. After analysing the results and in an attempt to mimic the tumour microenvironment we added the conditioned medium of endothelial cells (HMCS and HMCT), along with the peptide treatment, and observed if in these conditions the peptide would exhibit different results.

### Proliferation assay and cellular morphology analysis

To analyse cell proliferation in the HaCaT and HeLa cells, a growth curve was performed. To count the number of cultured cells, they were seeded at a concentration of 3 × 10^4^ in 1 mL of complete medium for 24 h. After this period, the medium was replaced with a serum‐free medium, with the purpose of maintaining the same cellular phase. After a further 24 h, this medium was replaced again with complete medium, with the addition of specific HMC and Ac2‐26 peptide, according to the experimental groups. The cells were analysed and counted at six different time points (2, 4, 24, 48, 72 and 120 h). The cellular morphology was evaluated with inverted microscopy, using an Olympus CKX41 (Olympus, Tokyo, Japan).

### Wound healing assay

The HaCaT and HeLa cells were distributed in 12‐well plates; after reaching adherence and confluence, a wound was made in the centre of the well. Subsequently, the cells were subdivided according to the experimental groups and analysed at 0, 2, 4, 24, 48, 72, and 120 h. The cellular motility was monitored with images obtained using a photographic camera coupled to the microscope. The wound areas were determined in six different microscopic fields and quantified using ‘imagej software (National Institute of Health ‐ NIH, Bethesda, MD, USA).

### Cytotoxicity and viability assay (MTS)

The cells were subdivide into the experimental groups and handled according to the manufacturer's protocol for the CellTiter 96^®^AQUeous One Solution Cell Proliferation Assay (Promega, Madison, WI, USA), which uses the tetrazolium compound [3‐(4,5‐dimethylthiazol‐2‐yl)‐5‐(3‐carboxymethoxyphenyl)‐2‐(4‐sulfophenyl)‐2*H*‐tetrazolium, inner salt; MTS], and an electron coupling reagent (phenazine ethosulfate). MTS reagent was added and absorbance at 490 nm was recorded using an ELISA plate reader. The analyses were made at the times of 2, 4, 24, 48, 72, and 120 h, and were evaluated by comparing the cellular viability across the experimental groups, as well as IC_50_ calculus (inhibitory concentration to 50% of the cells).

### Flow cytometry

The cells were subdivided into the experimental groups and handled according to the manufacturer's protocol for the FITC Annexin V Apoptosis Detection Kit I (BD Pharmingen™, São Paulo, SP, Brazil), to investigate viability, apoptosis, late apoptosis and necrosis, and utilised Guava Easy Cyte (Millipore, Barueri, SP, Brazil) equipment to perform the analysis.

### Selection of the genes

The genes were previously selected from potential markers in the tumour microenvironment, evaluated in the studies of 64, 65. The metabolic pathways from these potential markers were evaluated and some related genes were also chosen. The gene selections took into consideration those that were potentially involved in the tumour invasion and inflammatory processes.

The genes selected were: *COX2* (also known as prostaglandin‐endoperoxide synthase 2; *PTGS2*), *EP3* (or *PRGER3*), *EP4* (or *PTGER4*), *MMP2*,* MMP9* and TIMP metallopeptidase inhibitor 1 (*TIMP1*). The specific primers for each transcript were designed with primer3 (http://primer3.ut.ee/), as shown in Table 1.

**Table 1:** Primers for the studied genes

Oligonucleotide	Sequence
*COX2* anti‐sense	5′ AGAAGGCTTCCCAGCTTTTG 3′
*COX2* sense	5′ ATTCCCTTCCTTCGAAATGC 3′
*EP3* anti‐sense	5′ TCTCCGTGTGTGTCTTGCAG 3′
*EP3* sense	5′ AGCTTATGGGGATCATGTGC 3′
*EP4* anti‐sense	5′ CCAAACTTGGCTGATATAACTGG 3′
*EP4* sense	5′ CGAGATCCAGATGGTCATCTTAC 3′
*MMP2* anti‐sense	5′ CCGTCAAAGGGGTATCCATC 3′
*MMP2* sense	5′ AAGTCTGGAGCGATGTGACC 3′
*MMP9* anti‐sense	5′ ATTTCGACTCTCCACGCATC 3′
*MMP9* sense	5′ TTGTGCTCTTCCCTGGAGAC 3′
*TIMP1* anti‐sense	5′ TTTTCAGAGCCTTGGAGGAG 3′
*TIMP1* sense	5′ ACTGTTGGCTGTGAGGAATG 3′

### Quantitative real time PCR

The reactions were performed in the 7500 Fast Real‐Time PCR system thermocycler (Applied Biosystems, Foster City, CA, USA); all the reactions were carried out in a final volume of 20 μL with 100 ng of cDNA, SYBR^®^ Green PCR Master Mix, and 100 nm of each primer (F and R). Glyceraldehyde‐3‐phosphate dehydrogenase (*GAPDH*) was the best endogenous control gene when tested alongside actin beta (*ACTB*). The analyses were performed from the cycle threshold (*C*_t_) of each sample according to 66.

### Statistical analysis

All assays were performed with three independent experiments. prism version 6.0 (GraphPad Software Inc., San Diego, CA, USA) was used to perform all statistics. The Kolmogorov–Smirnov normality test was used to analyse distribution. Variance analysis (ANOVA) was used for between group comparisons, followed by the appropriate parametric or non‐parametric *post‐hoc* test. Statistical significance was set at a probability value of less than 0.05.

## Conflict of interest

The authors declare no conflict of interest.

## Author contributions

LTC designed and performed all experiments and wrote the manuscript. JP contributed to the analysis of the flux cytometry. BRC performed the experiments with the quantitative PCR. EHT contributed to writing the manuscript. SMO supported the experimental development and contributed to writing the manuscript. FCRL coordinated the project, and contributed to the experiments and writing of the manuscript.
